# Effect of Birth Weight on Muscle Strength and Body Composition in Young Adult Men and Women

**DOI:** 10.7759/cureus.86528

**Published:** 2025-06-22

**Authors:** Tomohiro Yasuda

**Affiliations:** 1 Exercise Physiology, Seirei Christopher University, Hamamatsu, JPN

**Keywords:** birth weight, body composition, muscle strength, skeletal muscle mass, young adults

## Abstract

Background and aim: Low birth weight (birth weight of less than 2500 g) among Japanese women has a negative impact on physical form and physical strength evaluation in young adulthood, but there are very few research reports on birth weight and physical form and strength in young adulthood. In addition, there may be differences in the impact of birth weight on physical form and physical strength in young adulthood between men and women, as the extreme desire to be thin that is common among young adult women in Japan is not seen among young adult men. This study aimed to examine the impact of birth weight on the morphology, body composition, and muscle strength of Japanese adult men and women.

Method: A total of 107 young adults (39 men and 68 women) participated in the study, and the Pearson product-moment correlation coefficients between birth weight and height, weight, BMI, skeletal muscle index (SMI), grip strength, knee extension strength, and chair stand test were examined. Multiple regression analysis was performed using the items that showed a significant relationship with birth weight as independent variables.

Results: The birth weight of women was significantly correlated with height, weight, skeletal muscle index (SMI), regional subcutaneous fat, regional skeletal muscle mass, grip strength, and knee extension strength. Multiple regression analysis revealed that only the skeletal muscle mass of the right leg was affected, and the coefficient of determination (R^2^) was 0.202 (p=0.01). However, no significant correlation was found between birth weight and any of the variables in men, so multiple regression analysis was not performed.

Conclusion: Unlike in men, the birth weight of women has an impact on their body composition, muscle strength, and physical form in young adulthood. In particular, the results suggest that birth weight may have a strong impact on lower-limb muscle mass and that women's birth weight may be a factor to consider when determining the risk of becoming bedridden in the future and its impact on the next generation.

## Introduction

The prevalence of low-birth-weight babies has been increasing annually since the 1980s, and in recent years, it has remained at approximately 10%, with no sign of a decrease [1-3]. Similarly, the prevalence of underweight young adult women aged 20-29 years has also been increasing since approximately 1980, and presently, approximately 20% of young adult women are underweight [4,5]. Recently, it has been reported that women of reproductive age in Japan have a strong desire to lose weight, and it is thought that the desire of young adult women to lose weight is contributing to the increase in low-birth-weight babies [6,7].

Yasuda reported that low birth weight (birth weight of less than 2500 g) among Japanese women has a negative impact on physical form and physical strength evaluation in young adulthood, but very few studies have reported on birth weight, body composition, and muscle strength in young adulthood [8]. In addition, because the extreme desire to be thin that is common among young adult women in Japan is not observed among young adult men, there may be sex differences in the impact of birth weight on the morphology, body composition, and muscle strength of young adults [9].

Both genetic and environmental factors are believed to influence physical characteristics, body shape, and muscle strength in young adults. For example, studies have reported that genetic and environmental factors have an equal influence on muscle strength [10]. Therefore, even if environmental factors influence children during early childhood and adolescence, birth weight is expected to have a significant impact on young adults.

In this study, the impact of birth weight on the morphology, body composition, and muscle strength of Japanese men and women in adulthood was examined. Young adults in Japan tend to be thin, with the lowest BMI among the Organization for Economic Co-operation and Development (OECD) countries for both men and women [11]. This is particularly pronounced among women, who are not only noted to be thin at levels comparable to those in poor countries, but also to have a strong desire to be thin, even among those who are thin [12]. Therefore, it would be very interesting to examine the differences between men and women by targeting Japanese who are characterized as thin in the OECD.

## Materials and methods

Participants

**Figure 1:**
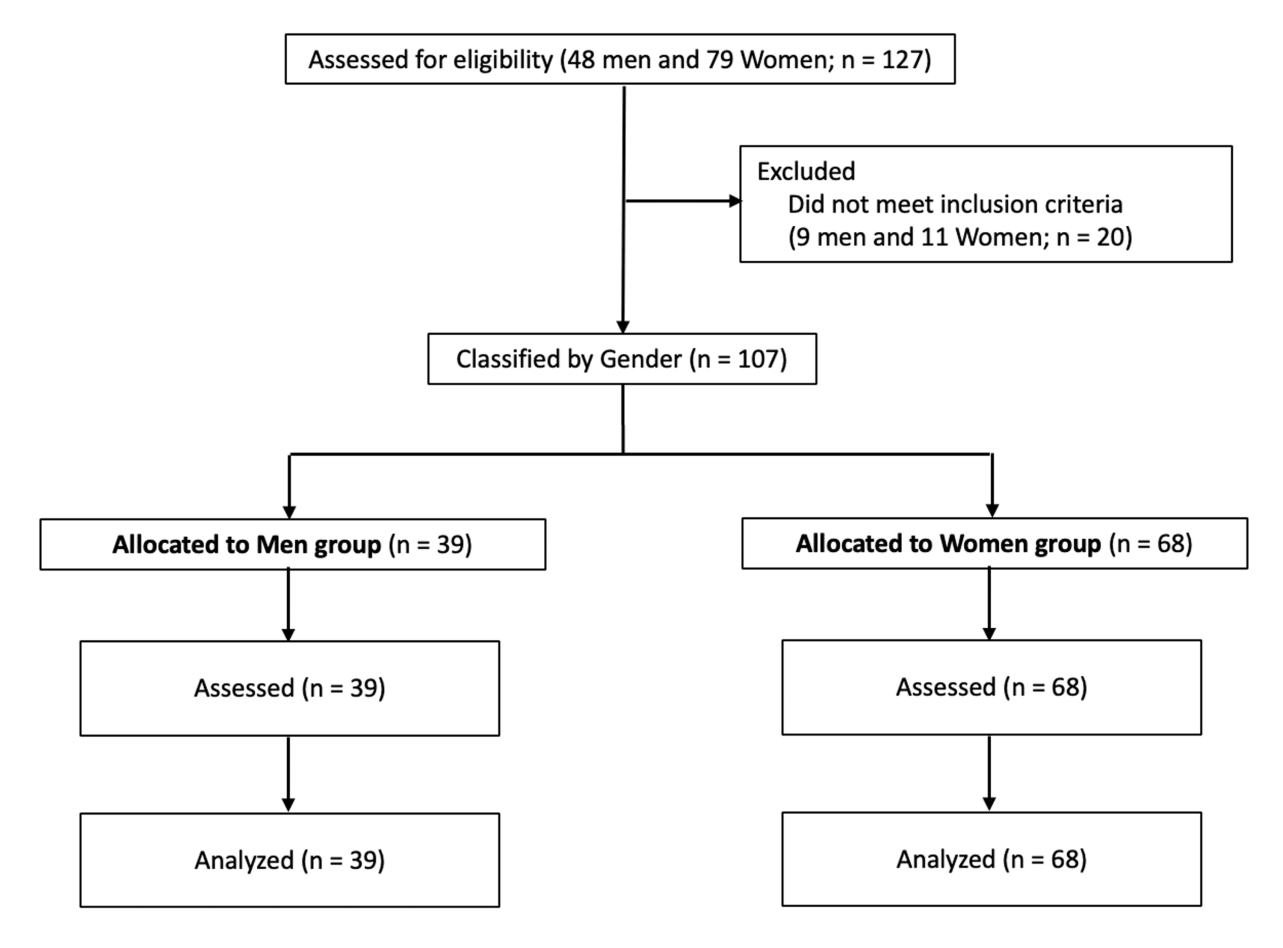
The flow diagram of the study sample.

Healthy young women were verbally invited to participate in the study, and 127 young Japanese adults aged 18-21 years (48 men and 79 women) were registered as potential subjects. Excluding subjects with unknown birth weight or incomplete measurements, data from 107 subjects (39 men and 68 women) were included in the analysis (Figure 1).

All participants were confirmed to have no chronic diseases (heart attack, angina pectoris, myocardial infarction, diabetes, cancer, stroke, etc.) and no history of musculoskeletal diseases or knee surgery on the basis of their annual health assessment. A written explanation of the purpose and safety of the study, as well as a questionnaire on lifestyle, was distributed to potential participants before informed consent was obtained. The study participants were medical undergraduates, approximately 90% of whom were classified as recreationally active and exercised at low-to-moderate intensity one-to-three times per week. The remaining 10% were members of athletic clubs (kendo, baseball, and volleyball clubs). All participants who fulfilled the criteria were included in the data analysis. The ethical principles of the Helsinki Declaration were adopted in this study. This study was approved by the Ethics Committee of Seirei Christopher University, and all participants provided written informed consent.

Muscle strength

A factory-calibrated hand dynamometer (TKK 5401; Tokyo, Japan: Takei Scientific Instruments) was used to assess the maximum voluntary isometric contraction of grip strength. All the participants were instructed to grip the dynamometer with their right hand in an upright position with the arm next to the trunk and the elbow extended to 180°. The handle of the dynamometer was set to a size that was comfortable for the participant to hold (i.e., the second joint of each finger fits inside the handle {Ed1}). Each participant completed two trials, and the highest value on each trial was used in the analysis [13-16]. The maximal voluntary isometric contraction (MVC) of the knee extensor muscles was measured using a knee extensor dynamometer (TKK 5710; Tokyo, Japan: Takei Scientific Instruments). The participants sat in a chair with their hip joint at an angle of approximately 85° (full extension=0°). The right ankle of each participant was secured to the strain gauge transducer with a chain strap (TKK 5002; Tokyo, Japan: Takei Scientific Instruments). After a warm-up consisting of maximum voluntary isometric contraction, the subjects were instructed to extend their knees to the maximum extent possible while keeping their knees bent at approximately 90 degrees (for 2-3 seconds). All the subjects performed two trials, and the highest value of each trial was used for analysis [17].

Body composition

Standing height was measured using a height meter in 0.5 cm increments, and body weight was measured using an electronic scale in 0.1 kg increments. Body mass index (BMI) was defined as weight/height^2^ (kg/m^2^). An InBody Analyzer (430; Seoul, Korea: BioSpace), which is a multifrequency bioelectrical impedance analyzer (BIA), was used according to the manufacturer's guidelines. BIA is used to estimate body composition on the basis of differences in conductivity caused by the different biological characteristics of different tissues. The body composition analyzer is based on a 4-electrode, eight-point contact system and measures the impedance of the legs, torso, and arms at three different frequencies (5, 50, and 250 kHz) for each body part. Participants were measured in a resting posture and in a standing position with their arms stretched out in front of them and their elbows straight; InBody automatically provided weight, body fat percentage, and skeletal muscle mass for each body part. The skeletal muscle index (SMI), defined as appendicular muscle mass (AMM) divided by height squared (kg/m²), was calculated by summing the muscle mass of both upper and lower extremities [14-16].

Statistical analyses

The results are presented as the means±standard deviations for all the variables. All the data were analyzed via JMP software version 12.0 (Tokyo, Japan: SAS Institute Inc.). Nonparametric statistical analysis (Wilcoxon signed-rank test) was applied to identify differences between the men and women groups when the data were not normally distributed. Statistical significance was defined as p<0.05. Correlation coefficients were calculated for birth weight with height, weight, BMI, skeletal muscle index (SMI), grip strength, knee extension strength, and performance in the chair stand test. Since the value of events per variable = 10 seems most prudent for regression analysis, 68 variable factors were acceptable for women [18]. In addition, the variance inflation factor (VIF) was used to determine the degree of multicollinearity of the i-th independent variable with other independent variables for all hierarchical regression models [19]. Multicollinearity between variables is defined as VIF being 10 or higher. In this study, birth weight showed a significantly high correlation coefficient, and since only the right arm was used for grip strength, when similar significant correlation coefficients were obtained for both sides, the right side was used. Based on the VIF results, we set up a stepwise multiple regression analysis (with a threshold value of p <0.05) to increase or decrease the variables and predicted birth weight and variable factors (standing height, body weight, SMM of the right arm, SMM of the trunk, and grip strength). As a result, the predictor variables, coefficients, and intercept coefficients were automatically extracted by JMP software. The sample size was estimated based on a prior power analysis [20]. This was done to detect the planned intervention effect (power 0.80, α=0.05, two-tailed test, effect size 0.95) to detect differences in weight between men and women. This was planned based on the results of a previous study comparing body shape and muscle strength in young men and women [21]. Using G*Power (Düsseldorf, Germany: Heinrich Heine University Düsseldorf) to calculate the sample size, it was determined that 19 participants of each gender were required. The number of participants in this study was set to sufficiently exceed this number (39 men and 68 women).

## Results

The physical characteristics and clinical data are listed in Table 1. There were significant differences in the values between the sexes, except for age (p=0.751), birth weight (p=0.693), and BMI (p=0.920). Among the participants, two men and three women were born with low birth weights (under 2500 g). In addition, in young adulthood, three men (7.7%) and 19 women (27.9%) whose SMIs were below the diagnostic criteria for sarcopenia were included.

**Table 1:** The physical characteristics in young adult men and women. *P-value <0.01 was statistically significant between men and women. Data are presented as mean (standard deviation). BMI: body mass index; SMI: skeletal muscle index

Variables	Men (n=39)		Women (n=68)	
	Mean (SD)	Range	Mean (SD)	Range
Birth weight (g)	3004 (378)	2326-4060	2973 (411)	1448-4124
Age (years)	18.8 (0.7)	18-20	18.7 (0.8)	18-21
Standing height (cm)	169.3 (6.4)*	158.5-184.0	157.5 (5.1)	146.5-170.0
Body weight (kg)	60.9 (9.6)*	44.2-86.6	52.6 (8.3)	39.2-94.2
BMI (kg/m^2^)	21.1 (2.5)	16.1-28.3	21.2 (3.2)	16.8-38.2
Morphological assessment				
Body fat (%)	14.4 (5.2)*	6.1-29.3	27.0 (6.0)	16.4-49.6
Fat mass (kg)	9.0 (4.2)*	3.2-20.3	14.6 (5.9)	7.4-46.7
Fat-free mass (kg)	32.6 (9.4)*	22.2-62.7	24.4 (7.1)	16.3-42.5
SMI (kg/m^2^)	7.68 (0.59)*	6.38-8.95	6.08 (0.59)	4.68-8.28
Fat mass				
Left arm (kg/m^2^)	0.54 (0.45)*	0.10-2.80	1.00 (0.55)	0.50-4.60
Right arm (kg/m^2^)	0.53 (0.49)*	0.10-3.00	0.98 (0.56)	0.50-4.60
Left leg (kg/m^2^)	1.74 (1.29)*	0.80-8.70	2.44 (0.87)	1.40-7.30
Right leg (kg/m^2^)	1.75 (1.31)*	0.80-8.80	2.45 (0.87)	1.40-7.30
Skeletal muscle mass				
Left arm (kg/m^2^)	2.63 (0.47)*	1.90-3.88	1.67 (0.30)	0.92-2.60
Right arm (kg/m^2^)	2.69 (0.48)*	2.00-3.81	1.72 (0.30)	1.09-2.60
Left leg (kg/m^2^)	8.35 (1.07)*	6.80-11.40	5.85 (0.71)	4.34-7.60
Right leg (kg/m^2^)	8.38 (1.09)*	6.80-11.60	5.87 (0.70)	4.39-7.60
Functional assessment				
Grip strength (kg)	41.3 (6.5)*	30.0-57.6	27.3 (4.0)	19.1-34.6
Knee extension (kg)	45.0 (10.0)*	26.3-77.0	28.8 (5.7)	18.3-45.5

The birth weight of women was significantly correlated with height, weight, SMI, regional subcutaneous fat, regional skeletal muscle mass, grip strength, and knee extension strength (Table 2). Multiple regression analysis revealed that only the skeletal muscle mass of the right leg was affected, and the coefficient of determination (R^2^) was 0.202 (p=0.01). However, no significant correlation was found between birth weight and any of the variables in men, so multiple regression analysis was not performed (Table 2).

**Table 2:** Pearson’s correlation coefficients between birth weight and variable data in young adult men and women. *P-value <0.01 was statistically significant. **P-value <0.05 was statistically significant. BMI: body mass index; SMI: skeletal muscle mass index

Variables	Men (n=39)		Women (n=68)	
	Birth weight	p-Value	Birth weight	p-Value
Standing height	0.197	0.23	0.426*	0.001
Body weight	0.195	0.255	0.377*	0.002
BMI	0.15	0.384	0.218	0.074
Percentage body fat	0.144	0.396	0.248**	0.041
Fat mass	0.194	0.251	0.278**	0.022
Fat-free mass	0.142	0.403	-0.047	0.702
SMI	0.05	0.769	0.289**	0.017
Fat mass, left arm	0.082	0.631	0.199	0.104
Fat mass, right arm	0.094	0.582	0.211	0.084
Fat mass, trunk	0.125	0.459	0.309**	0.01
Fat mass, left leg	0.067	0.692	0.275**	0.023
Fat mass, right leg	0.064	0.705	0.275**	0.023
SMM, left arm	0.202	0.231	0.351*	0.003
SMM, right arm	0.184	0.276	0.338*	0.005
SMM, trunk	0.206	0.222	0.377*	0.002
SMM, left leg	0.093	0.585	0.443*	0.001
SMM, right leg	0.102	0.547	0.449*	0.001
Grip strength	0.143	0.386	0.395*	0.001
Knee extension strength	0.11	0.505	0.258**	0.035

## Discussion

The main findings of this study are as follows. First, birth weight had a greater relevance on young adult women than on young adult men, even though there was no difference in birth weight or BMI during adolescence between the sexes. Second, the relevance of birth weight in women may extend to their body shape, body composition, and physical strength in adolescence and beyond.

The standing height, body weight, BMI and grip strength of the research participants (19-year-old men: 169.3 cm, 60.9 kg, 21.1 kg/m^2^, 41.3 kg; women: 157.5 cm, 52.6 kg, 21.2 kg/m^2^, 27.3 kg) are broadly similar to the standard values for Japanese people's physical form and physical ability (men: 171.1 cm, 63.0 kg, 21.4 kg/m^2^, 45.8 kg; 19-year-old women: 158.7 cm, 52.2 kg, 20.7 kg/m^2^, 28.4 kg) (Table 1) [22]. In addition, the birth weights of Japanese men and women in 2022 were 3050 g and 2960 g, respectively, which are very close to the values of the participants in this study (men: 3004 g, women: 2973 g) (Table 1) [23]. Thus, the participants recruited for this study were representative of the public population of healthy young adult men and women in Japan.

A previous study classified the birth weights of women and reported that low-birth-weight infants had significantly lower height, lower-limb power, SMI, and grip strength in young adulthood [8]. In this study, there were only three low-birth-weight infants (under 2500 g), so it was difficult to evaluate them in groups; therefore, the relationship with each item was examined for all women. In this assessment, there were significant correlations between standing height and body weight, the SMI, body composition (fat mass and skeletal muscle mass), grip strength, and lower-limb muscle strength, and the results were in line with those of a previous study in which participants were divided into groups on the basis of these variables. As reported in previous studies, it is important to maximize the functional capacity of muscles during childhood, which is thought to be effective as a measure against the future development of conditions that result in bedridden status (e.g., reducing sarcopenia) [24]. It has also been suggested that low birth weight is associated with a significant decrease in muscle fibers at the age of 68-76 years. Taken together, the results of these studies and the present study suggest that even in samples where there are few low-birth-weight infants, women's birth weight is related with their physical form, body composition, and muscle strength later in life, and there is a possibility that they will have a high rate of sarcopenia in the future. Therefore, even if a woman does not fall into the category of low birth weight, it is necessary to be aware of birth weight and pay attention to health promotion, including the prevention of sarcopenia, because the effects on the body persist after young adulthood.

Unlike the women in this study, the birth weight of men was not significantly correlated with any of the other variables. Although previous studies have shown a significant correlation between birth weight and grip strength in both older adults and at age 31, after adjusting for sex differences, the number of related studies remains limited [25,26]. The results of the present study cannot be seen as the same trend as in previous studies, and there have been few clear research findings to date that show this difference. The coefficients of variation in birth weight, age at young adulthood, height, weight, and BMI (12.6% vs. 13.8%, 4.0% vs. 4.1%, 3.8% vs. 3.2%, and 12.1% vs. 15.0%, respectively) for the men and women in the study were also not significantly different between the two groups. Therefore, it may be necessary in the future to focus on the possibility of significant differences in birth weight and young adult morphology, body composition, and muscle strength, including inherent and acquired influences. Exploring the potential biological mechanisms underlying these sex differences (e.g., differences in growth hormone sensitivity, the effects of estrogen/testosterone on muscle development, etc.), in addition to behavioral or environmental explanations, may be beneficial.

The present study has several limitations. First, the participants were young Japanese adults, which limited their ethnicity, age, and physical characteristics. Second, the proportion of low-birth-weight babies (under 2500 g) in this study was two men (5.1%) and three women (4.4%), which is approximately half the Japanese average of 9.4%. Although participants were recruited randomly, the cause of this is unclear. This study has several limitations, including its cross-sectional design, which prevents determination of causality, and a notably small sample size. Future robust studies with large sample sizes are needed to validate the present results.

## Conclusions

The results of this study showed that, unlike in men, the birth weight of women is related to their body composition, muscle strength, and physical form in young adulthood. In particular, the results suggest that birth weight may have a strong impact on lower-limb muscle mass.
